# The Current Situation of Hypertension among Rural Minimal Assurance Family Participants in Liaoning (China): A Cross-Sectional Study

**DOI:** 10.3390/ijerph13121199

**Published:** 2016-12-02

**Authors:** Yintao Chen, Shasha Yu, Shuang Chen, Xiaofan Guo, Yuan Li, Zhao Li, Yingxian Sun

**Affiliations:** Department of Cardiology, the First Hospital of China Medical University, Shenyang 110001, China; chenyintao1990@126.com (Y.C.); yidasasa@foxmail.com (S.Y.); 13654004558@126.com (S.C.); guoxiaofan1986@foxmail.com (X.G.); wxy13196303669@163.com (Y.L.); zhaolisy@outlook.com (Z.L.)

**Keywords:** subsistence security system, minimum living allowance, hypertension, socioeconomic status

## Abstract

In China, the prevalence of hypertension is increasing and is showing an epidemic accelerating trend. However, there is a lack of studies reporting the hypertension status of rural residents with minimum living allowances. We performed a cross-sectional study including 11,435 (5285 men and 6150 women) from the general population aged ≥35 years in the Liaoning Province of China from 2012 to 2013, of which 1258 (11.0%) participants came from minimal assurance families. Anthropometric measurements, laboratory examinations and self-reported lifestyle factor information were collected by trained personnel. Multivariate logistic regression was used to detect the association between socioeconomic status (SES) and the risk of hypertension. We found that the prevalence of hypertension was as high as 61.9% in participants from minimal assurance families and the odd ratio for hypertension was 1.32 (95% CI: 1.15–1.52). The awareness, treatment, and control rates among treated hypertensive participants did not increase with higher level of income and education. In the total sample, the lower income levels increased the risk for hypertension, but education didn’t show a significant association with hypertension. Thus, there is a severe hypertension situation in the Liaoning rural population of minimal assurance families, which need more attention and prevention and control measures for hypertension.

## 1. Introduction

Hypertension is a global public health problem affecting nearly one billion people worldwide [1,2]. In China, the prevalence of hypertension is increasing and showing a trend of becoming an accelerating epidemic [3,4]. Previous studies have reported that the prevalence of hypertension was higher in urban than in rural communities [5,6,7], but some recent studies showed that the prevalence of hypertension in rural areas exceeded that in the urban districts in China [4]. Northern China is a high hypertension incidence area, and the northern rural areas also face a grim hypertension status. The prevalence of hypertension has increased dramatically from 37.8% in 2004–2005 to 51.1% in 2012–2013 in rural Northeast China [8,9]. Studies have reported that obesity, diabetes, dyslipidemia, and hyperuricemia are becoming risk factors in rural China [9]. Because of the huge rural population base, a small prevalence growth may lead to serious public health problems [7]. Considering the disparities in ethnicity, regions and other factors influencing the prevalence of hypertension, various targeted interventions are needed that span the population and focus on vulnerable groups [7].

The subsistence security system is a basic living security policy in China, which aims to provide residents with a minimum living allowance. Its target populations are those residents who are living on the edge of poverty or whose per capita household income is lower than local minimum income standard, and someone losing job temporarily or becoming disabled. It is an essential method to solve the problem of food and clothing of poor residents and guarantee their basic life rights. There is a large wealth gap between the urban and rural areas in China, so rural residents with the minimum living allowance are in a worse living environment.

In rural areas across China, the subsistence security system has covered a large portion of the population, from 35.66 million in 2007 to 53.88 million in 2013 [10]. Usually, they are people who are older, solitary, have low-income or even no income, disabled, out of work or valetudinarian. Thus, the residents with minimum living allowance are a special population who are of overall relatively low socioeconomic status (SES). However, until now there has been a lack of studies reporting on the hypertension or health status of rural residents with minimum living allowance. The present study focused on a special population, rural residents from minimal assurance families, and aimed to study the hypertension status of the group, including hypertension prevalence, awareness, treatment and control, and their quality of life (QOL). We expect that more attention must be paid to cardiovascular diseases, including hypertension, among the rural population with minimum living allowance.

## 2. Materials and Methods

### 2.1. Study Population

From July 2012 to August 2013, we selected a representative general sample aged ≥35 years to describe the prevalence, incidence and natural history of cardiovascular risk factors in rural areas of Liaoning Province, which is located in Northeast China. The study adopted a multi-stage, stratified randomly cluster-sampling scheme. In the first stage, three counties (Dawa, Zhangwu, and Liaoyang County) were selected from the eastern, southern, and northern regions of Liaoning Province. In the second stage, one town was randomly selected from each county (a total of three towns). In the third stage, 8–10 rural villages from each town were randomly selected (a total of 26 rural villages). All the eligible permanent residents aged ≥35 years from each village were invited to participate in the study (a total of 14,016 participants). Of those, 11,956 participants agreed and completed the present study for a response rate of 85.3%. The study was approved by the Ethics Committee of China Medical University (Shenyang, China) (AF-SDP-07-1, 0-01). All procedures were performed in accordance with the ethical standards. Written consent was obtained in all participants after they had been informed of the objectives, benefits, medical items and confidentiality agreement of personal information. If the participants were illiterate, we obtained written informed consents from their proxies. In this report, we used data of baseline and only participants with a complete set of data regarding the variables analyzed in the study were included, giving a final sample size of 11,435 (5285 men and 6150 women).

### 2.2. Data Collection and Measurements

Data on demographic characteristics, lifestyle risk factors, family income, medical history and medical expenses were obtained using a standardized questionnaire during face-to-face interviews performed by cardiologists and trained nurses during a single clinic visit. Before the survey was performed, we invited all eligible investigators to attend organized training. The training contents included the purpose of this study, how to administer the questionnaire, the standard methods of measurement, the importance of standardization, and the study procedures. A strict test was applied after this training, and only those who scored perfectly on the test were selected as investigators. During data collection, our inspectors received further instructions and support. There was a central steering committee with a subcommittee for quality control.

Marital status was categorized in two groups: married or living with partner, and unmarried, divorced or widowed. Educational level was divided into primary school or below, middle school and high school or above. Family income was classified as ≤5000, 5000–20,000 and >20,000 China Yuan (CNY)/year. The smoking and alcohol consumption status were also surveyed. Current smokers were defined as people who were currently smoking. Alcohol drinkers were defined as people who were currently drinking. Occupational physical activity, including the farm work, was classified as low, moderate and heavy physical load. The information about participation in the subsistence security system was acquired with the question “is your family under the local rural subsistence security system”.

According to American Heart Association protocol, blood pressure (BP) was measured three times at 2-min intervals after at least 5 min of rest using a standardized automatic electronic sphygmomanometer (HEM-741C; Omron, Tokyo, Japan). The calibration of the Omron device was checked every month using a standard mercury sphygmomanometer by two doctors according to the British Hypertension Society protocol [11]. The participants were advised to avoid caffeinated beverages and exercise for at least 30 min before the measurement. During the measurement, the participants were seated with the arm supported at the level of the heart. The mean of three BP measures was calculated and used in all analyses.

Weight and height were measured to the nearest 0.5 kg and 0.1 cm respectively with the participants in light weight clothing and without shoes. Waist circumference (WC) was measured at the midpoint between the lower rib and upper margin of the iliac crest using a non-elastic tape (to the nearest 0.1 cm), with the participants standing at the end of normal expiration.

Fasting blood samples were collected in the morning after at least 12 h of fasting for all participants. Blood samples were obtained from an Antecubital vein into Vacutainer tubes containing ethylenediamine tetraacetic acid (EDTA). Serum was subsequently isolated from the whole blood, and all serum samples were frozen at −20 °C for testing at a central, certified laboratory. Fasting plasma glucose (FPG), total cholesterol (TC), low-density lipoprotein cholesterol (LDL-C), high-density lipoprotein cholesterol (HDL-C), triglyceride (TG), Serum uric acid(SUA) and other routine blood biochemical indexes were analyzed enzymatically on an AU640 autoanalyzer (Olympus, Kobe, Japan). All laboratory equipment was calibrated and blinded duplicate samples were used.

### 2.3. Definitions

According to the Seventh Report of the Joint National Committee on Prevention, Detection, Evaluation, and Treatment of High Blood Pressure (JNC 7) [12], hypertension is defined as systolic blood pressure (SBP) 140 mmHg or greater, diastolic blood pressure (DBP) 90 mmHg or greater, and/or use of antihypertensive medications. The newly-diagnosed hypertension was defined as an average SBP of at least 140  mmHg, average DBP of at least 90  mmHg in this survey without any prior diagnosis of hypertension. Awareness of hypertension was defined as self-reporting of any prior diagnosis of hypertension by a health care professional among the population defined as having hypertension. Treatment of hypertension was defined as use of a prescription medication for management of hypertension at the time of the interview. Control of hypertension was defined as pharmacologic treatment of hypertension associated with an average SBP < 140 mmHg and an average DBP < 90 mmHg [13].

The quality of life (QOL) was measured with the mainland Chinese version of the WHOQOL-BREF [14,15] which is a self-report inventory with 26 original items. The items fall into four domains: the physical health (seven items), the psychological health (six items), the social relationships (three items) and the environment (eight items), together with two items measuring overall QOL and general health [16]. The scale has demonstrated good internal consistency with Cronbach’s alpha ranging from 0.67–0.81 for each domain. Each item is answered on a 5-point response scale, and the range of scores is form 4 to 20 after calculation, with higher scores indicating better QOL.

Dyslipidemia was defined according to the National Cholesterol Education Program Third Adult Treatment Panel (ATP III) criteria [17]. High TC was defined as TC ≥ 6.21 mmol/L (240 mg/dL). Low HDL-C was defined as HDL-C < 1.03 mmol/L (40 mg/dL). High LDL-C was defined as LDL-C ≥ 4.16 mmol/L (160 mg/dL). High TG was defined as ≥2.26 mmol/L (200 mg/dL). Diabetes mellitus was diagnosed according to the WHO criteria [18]: FPG ≥ 7 mmol/L (126 mg/dL) and/or being under treatment for diabetes.

### 2.4. Statistical Analysis

Differences among categories in the baseline characteristics were evaluated using Student’s *t*-test or Pearson chi-squared test as appropriate. The associations of minimal assurance family and socioeconomic status with hypertension were tested using univariate and multivariate logistic regression models, with odds ratios (ORs) and 95% confidence intervals (CIs) calculated. All statistical analyses were performed using SPSS version 22.0 software (IBM Corp., Armonk, NY, USA), and *p* < 0.05 indicated statistical significance.

## 3. Results

The characteristics of all the participants are shown in Table 1. 1258 (11.0%) participants came from minimal assurance families. Compared with ordinary family participants, those from minimal assurance families had lower income levels, education levels, occupational physical activity and prevalence of married/cohabiting, but they showed significantly higher mean SBP (147.9 ± 25.5 mmHg, *p* < 0.001). Although in the minimal assurance families groups more than 50% had an annual income between 5000 and 20,000 CNY/year, there were only 10.8% whose family annual income was over 20,000 CNY, and just 4.5% of the participants from minimal assurance families had high school and above educational level. Nevertheless they spent much more money on medicine and health care per year than participants from ordinary families.

**Figure 1:**
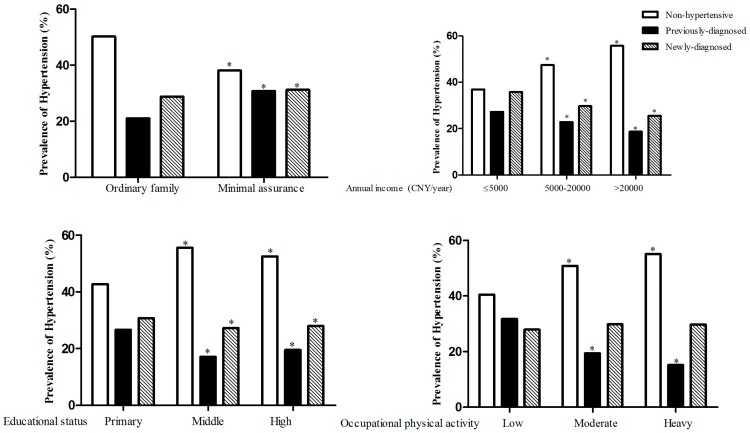
Prevalence of hypertension according to minimal assurance and socioeconomic status. * *p* < 0.05.

As shown in Figure 1, the prevalence of hypertension was higher in minimal assurance families, both for previously-diagnosed (30.8%) and newly-diagnosed hypertension (31.2%). With increased income level, educational status and occupational physical activity, the prevalence of hypertension decreased obviously, except for the newly-diagnosed hypertension patients in the occupational physical activity groups.

**Figure 2:**
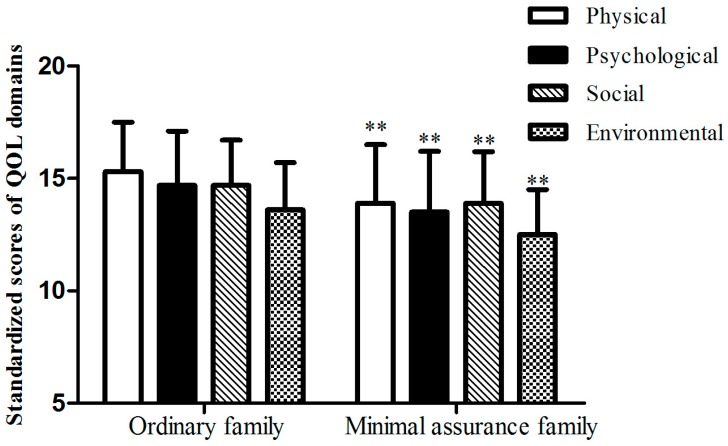
The mean scores of QOL domains in participants from minimal assurance families. ** *p* < 0.001.

Figure 2 shows the mean scores of QOL domains based on family status. Scores of each domain decreased in the minimal assurance family members. In contrast to ordinary families, the quality of life in all aspects, including the physical, psychological, social and environmental domains, was lower in participants from minimal assurance families.

Overall, 5845 (51.1%) participants were diagnosed with hypertension, including previously-diagnosed (2523, 22.1%) and newly-diagnosed (3322, 29.1%). The prevalence of hypertension is as high as 61.9% in participants from minimal assurance families. In participants from minimal assurance families with high school and above educational level, the prevalence of hypertension was 71.9%. Furthermore, the awareness, treatment, and control rates among treated hypertensive participants did not increase with higher level of income and education. As shown in Table 2, 49.8% (388/779) of minimal assurance family members with hypertension were aware of their diagnosis. Of those with hypertension, 38.4% (299/779) were under medical treatment, but only 13.7% (41/299) became normotensive with treatment, which was significantly lower than participants from ordinary families.

The associations between the socioeconomic status (SES) and hypertension are presented in Table 3.

Both in unadjusted (OR = 1.64, 95% CI: 1.46–1.85) and adjusted (OR = 1.27, 95% CI: 1.10–1.46) models, the minimal assurance family participants were strongly associated with a greater risk of hypertension. Compared with the highest income group, population with annual income less than 5000 CNY/year (OR = 1.19, 95% CI: 1.03–1.38) and between 5000 and 20,000 CNY/year (OR = 1.17, 95% CI: 1.07–1.29) had positive association with hypertension. In minimal assurance family group, only participants with low occupational physical activity also increased risk for hypertension (OR = 1.38, 95% CI: 1.01–1.87). But education didn’t show significant association with hypertension.

## 4. Discussion

It was astonishing to find that the prevalence of hypertension in participants from minimal assurance families was as high as 61.9%, which was much higher than the prevalence of the total sample and other reported data [19,20]. In hypertensive patients of the minimal assurance groups, 49.8% were aware of their hypertension diagnosis and 38.4% were taking treatment action, both of which were higher than the participants from ordinary families, but only 13.7% of treated hypertension patients were controlled in the minimal assurance groups. Increased income level and educational status didn’t increase the prevalence of awareness, treatment and control. An international study [21] reported that greater education was associated with greater awareness, treatment and rates of control in low-income countries only, and China was grouped in low-middle-income country in that study. Another national study [22] from China also surprisingly observed that in rural areas people with higher income had comparatively lower levels of awareness, treatment, and control rates, which was different with urban residents. Some researchers contributed the results to the rapid Chinese economic development and the imbalance between unhealthy behaviours or lifestyles and people’s economic status [22].

Theoretically, with modern lifestyles SES should have a negative relationship with the risk of hypertension [23]. In urban and developed countries the education and income were inversely associated with hypertension [24], although a systematic review [23] concluded that the association of educational status with hypertension was diametrically opposite in East Asian rural populations and South Asian rural populations, and in the latter a positive association was observed. Similarly, higher income was positively associated with hypertension in South Asia and teachers and bankers of Ethiopia [25] whereas no association was detected in East Asia and Africa [23]. However, several other studies conducted in developing countries also found out that education and income are inversely associated with the odds of developing hypertension [26,27], so the association of SES with hypertension differs according to geographical region. Previously, researchers proposed this association might be influenced by the phenomenon of epidemiological transition [28] and the society in China is experiencing comprehensive and profound social changes, so the association of SES with hypertension has no consistent trend at present and needs further research.

The present study found that participants from minimal assurance families had high risk levels for hypertension. There was not a great difference in economic status among the rural population, as in the minimal assurance families more than 50% of the people also had an annual income between 5000 and 20,000 CNY/year, but the hypertension prevalence in minimal assurance families is near to that of the lowest income group (61.9% vs. 63.1%). Perhaps the income levels in the minimal assurance families were closer, and an inverse association of income and hypertension couldn’t be found in the minimal assurance family group. Thus, only using income level to evaluate the risk of hypertension might underestimate the hypertension status in minimal assurance families. Such a high prevalence of hypertension in minimal assurance families might result from more negative factors they had. Usually they had not only low income, but also low education and low QOL. Indeed, more large sample studies or longitudinal studies are needed to elucidate the real causes.

The minimal assurance families are a special population in Chinese rural areas, and they represent the people at the bottom of society. In the present study, nearly two-thirds (65.3%) of the minimal assurance family members only had a primary school educational degree, or even never had any formal education. Maybe most participants from minimal assurance families were older and unemployed, so more than half of those had low occupational physical activity and nearly one-thirds of those had a very low income. When using the WHOQOL-BREF instrument to evaluate the QOL, we also found that all the mean scores of physical, psychological, social and environmental domains were obviously lower in minimal assurance families than ordinary families. Thus, the overall living standards of participants in minimal assurance families were unsatisfactory, just like other low-income populations of other studies [29,30]. What’s worse, this population are always living with chronic diseases or disabled family members, which add a heavier burden on the family, so they are fighting with diseases and often deal with doctors, so perhaps there are higher prevalences of awareness and treatment in participants from minimal assurance families. However, possibly because of poor income and compliance, control in minimal assurance participants receiving treatment was very poor. They have not enough ability to pay for the combination therapies that are required to achieve blood pressure control [31].

Hypertension-related diseases and complications were a major burden in high- and low-income countries alike, responsible for around 7 million deaths annually [32]. In addition, cardiovascular disease, including both stroke and heart disease, is now the leading cause of death among Chinese adults [33]. However it’s easy for patients to neglect the existence of hypertension and it’s hard to keep taking anti-hypertensive drugs every day. Then, this results in poor awareness, treatment, and control rates of hypertension. We found such a severe situation of hypertension in Chinese rural population of minimal assurance families, which had extremely high prevalence of hypertension and low control rates. If we just grouped them by income or education levels, this grim situation of hypertension might be overlooked or underestimated. Thus, the government and public health departments should pay more attention to the hypertension administration of rural populations rather than just ensuring their basic income, especially the population of minimal assurance families. In other countries, there are also some similar allowance policies to ensure the basic life of those bottom populations. Cardiovascular health also needs more attention and studies helping prevention and control of hypertension worldwide.

The major limitation of the study was that it was a cross-sectional analysis which only allows assessment of associations, but not inferences with regard to causality. It will be necessary to confirm the associations of SES and minimum living allowance with hypertension among the Chinese population in future longitudinal studies and reveal the causes, and replication of the results in further investigations with large-scale population are necessary before firm conclusions can be drawn. Second, new-hypertension diagnosis was based on blood pressure measurement on one occasion only, which may have resulted in some participants being incorrectly classified into blood pressure categories. Repeated measurements for hypertension should be encouraged in subsequent studies. Third, some data such as smoking and drinking status was acquired by asking questions. Although the investigators had been trained strictly beforehand, some recall bias was inevitable, so the frequency of smoking and number of cigarettes, important risk factors, were not collected completely in this study.

## 5. Conclusions

In conclusion, the present study reported a severe situation of hypertension in a northeast Chinese rural population of minimal assurance families, which had extremely high prevalence of hypertension and low control rates. In the total sample, less income increased the risk for hypertension, but this association might not exist in the minimal assurance family group. If we just grouped the population by SES levels, this grim situation of hypertension in minimal assurance families might be overlooked or underestimated. Thus, the northeast rural population with minimum living allowance should be paid more attention for their hypertension and cardiovascular diseases and other more effective strategies taken.

## Figures and Tables

**Table 1:** Sample characteristics of the minimal assurance families.

Characteristic	Ordinary Family	Minimal Assurance Family	*p* Value
Age, years	53.3 ± 10.3	57.8 ± 11.4	<0.001
Gender			0.008
Male	4659 (45.8)	626 (49.8)	
Female	5518 (54.2)	632 (50.2)	
Married/cohabiting	9281 (91.2)	985 (78.3)	<0.001
Annual income (CNY/year)			<0.001
≤5000	1047 (10.3)	376 (29.9)	
5000–20,000	5485 (53.9)	746 (59.3)	
>20,000	3645 (35.8)	136 (10.8)	
Educational status			<0.001
Primary school or below	4879 (47.9)	821 (65.3)	
Middle school	4280 (42.1)	380 (30.2)	
High school or above	1018 (10.0)	57 (4.5)	
Occupational physical activity			<0.001
Low	3549 (34.9)	649 (51.6)	
Moderate	1970 (19.4)	216 (17.2)	
Heavy	4658 (45.8)	393 (31.2)	
Current smoking	3556 (34.9)	478 (38.0)	0.032
Current drinking	2330 (22.9)	258 (20.5)	0.056
Body mass index, kg/m^2^	24.8 ± 3.6	24.5 ± 4.0	0.007
waist circumference, cm	82.5 ± 9.8	82.1 ± 10.3	0.241
LDL-cholesterol, mmol/L	2.9 ± 0.8	3.0 ± 0.8	0.002
HDL-cholesterol, mmol/L	1.4 ± 0.4	1.4 ± 0.4	0.006
Triglycerides, mmol/L	1.6 ± 1.5	1.7 ± 1.7	0.537
Total cholesterol, mmol/L	5.2 ± 1.1	5.3 ± 1.1	0.056
Fasting glucose, mmol/L	5.9 ± 1.6	6.0 ± 1.8	0.241
Systolic blood pressure, mmHg	141.0 ± 23.0	147.9 ± 25.5	<0.001
Diastolic blood pressure, mmHg	81.9 ± 11.7	83.1 ± 12.4	0.001
Serum uric acid, mmol/L	291.8 ± 84.4	289.9 ± 84.5	0.451
Family medical expenses (CNY/year) ^a^	4411.0 (2000.0)	7990 (3000.0)	<0.001

Data are expressed as the mean SD or as n (%). ^a^ Values are mean (median). *p*-values represent the result of standard *T* test or Pearson chi-square test to detect differences between the groups.

**Table 2:** Prevalence, awareness, treatment, and control of hypertension in rural adults of different socioeconomic status.

Characteristic	Prevalence	Awareness	Treatment	Control
**Ordinary Family**	5066 (49.8)	2153 (42.5)	1548 (30.6)	310 (20.0)
Annual income (CNY/year)				
≤5000	645 (61.6)	271 (42.0)	203 (31.5)	46 (22.7)
5000–20,000	2825 (51.5)	1205 (42.7)	860 (30.4)	166 (19.3)
>20,000	1596 (43.8)	677 (42.4)	485 (30.4)	98 (20.2)
*p* values	<0.001	0.954	0.863	0.557
Educational status				
Primary school or below	2726 (55.9)	1260 (46.2)	913 (33.5)	176 (19.3)
Middle school	1870 (43.7)	700 (37.4)	495 (26.5)	107 (21.6)
High school or above	470 (46.2)	193 (41.1)	140 (29.8)	27 (19.3)
*p* values	<0.001	<0.001	<0.001	0.563
Occupational physical activity				
Low	2046 (57.7)	1084 (53.0)	836 (40.9)	158 (18.9)
Moderate	950 (48.2)	376 (39.6)	257 (27.1)	53 (20.6)
Heavy	2070 (44.4)	693 (33.5)	455 (22.0)	99 (21.8)
*p* values	<0.001	<0.001	<0.001	0.456
**Minimal assurance family**	779 (61.9)	388 (49.8)	299 (38.4)	41 (13.7)
Annual income (CNY/year)				
≤5000	253 (67.3)	119 (47.0)	91 (36.0)	16 (17.6)
5000–20,000	452 (60.6)	233 (51.5)	180 (39.8)	23 (12.8)
>20,000	74 (54.4)	36 (48.6)	28 (37.8)	2 (7.1)
*p* values	0.015	0.505	0.598	0.316
Educational status				
Primary school or below	539 (65.7)	263 (48.8)	206 (38.2)	27 (13.1)
Middle school	199 (52.4)	106 (53.3)	78 (39.2)	13 (16.7)
High school or above	41 (71.9)	19 (46.3)	15 (36.6)	1 (6.7)
*p* values	<0.001	0.504	0.943	0.53
Occupational physical activity				
Low	455 (70.1)	256 (56.3)	212 (46.6)	33 (15.6)
Moderate	125 (57.9)	53 (42.4)	36 (28.8)	4 (11.1)
Heavy	199 (50.6)	79 (39.7)	51 (25.6)	4 (7.8)
*p* values	<0.001	<0.001	<0.001	0.316

Pearson chi-square test was used to detect differences among the socioeconomic status groups.

**Table 3:** Multiple regression analyses of hypertension and socioeconomic status.

	Total Sample		Ordinary Family		Minimal Assurance Family	
	Wald	OR (95 % CI)	Wald	OR (95 % CI)	Wald	OR (95 % CI)
Model 1						
Minimal assurance family ^a^	65.13	1.64 (1.46–1.85) *	- **	-	-	-
Model 2						
Minimal assurance family ^a^	10.04	1.27 (1.10–1.46) *	-	-	-	-
Annual income (CNY/year) ^b^						
≤5000	5.18	1.19 (1.03–1.38) *	3.32	1.17 (0.99–1.38)	0.81	1.24 (0.78–1.98)
5000–20,000	11.18	1.17 (1.07–1.29) *	9.30	1.16 (1.06–1.28) *	0.85	1.22 (0.80–1.87)
Educational status ^c^						
Primary school or below	0.2	0.97 (0.83–1.13)	0.10	0.97 (0.83–1.14)	2.32	0.59 (0.30–1.16)
Middle school	0.001	1.00 (0.86–1.16)	0.18	1.03 (0.89–1.21)	4.16	0.49 (0.24–0.97) *
Occupational physical activity ^d^						
Low	3.4	1.10 (1.00–1.22)	1.51	1.07 (0.96–1.19)	4.17	1.38 (1.01–1.87)
Moderate	0.13	1.02 (0.91–1.14)	0.05	1.01 (0.90–1.14)	0.31	1.12 (0.76–1.64)

Model 1 was unadjusted; model 2 was adjusted for age, sex, BMI, marital status, current smoking, current drinking, serum uric acid, dyslipidaemia, diabetes. The reference groups were ^a^ ordinary family, ^b^ annual income above 20000 CNY/year, ^c^ high school or above, and ^d^ heavy occupational physical activity, respectively. OR, odds ratio; CI, confidence interval. * *p* < 0.05. ** The short dashes meaned that the variables didn’t be introduced into the regression model.
